# Supplementary material for: Direct measurements of ice-shelf flexure caused by surface meltwater ponding and drainage
Source: Nat Commun. 2019 Feb 13;10:730. doi: 10.1038/s41467-019-08522-5 (PMC6374411; doi:10.1038/s41467-019-08522-5)
Supplement: Supplementary file 1 — Supplementary Information [file 41467_2019_8522_MOESM1_ESM.docx]

**Direct Measurements of Ice-Shelf Flexure caused by Surface Meltwater Ponding and Drainage**

Banwell *et al*.

**Supplementary Information**

**
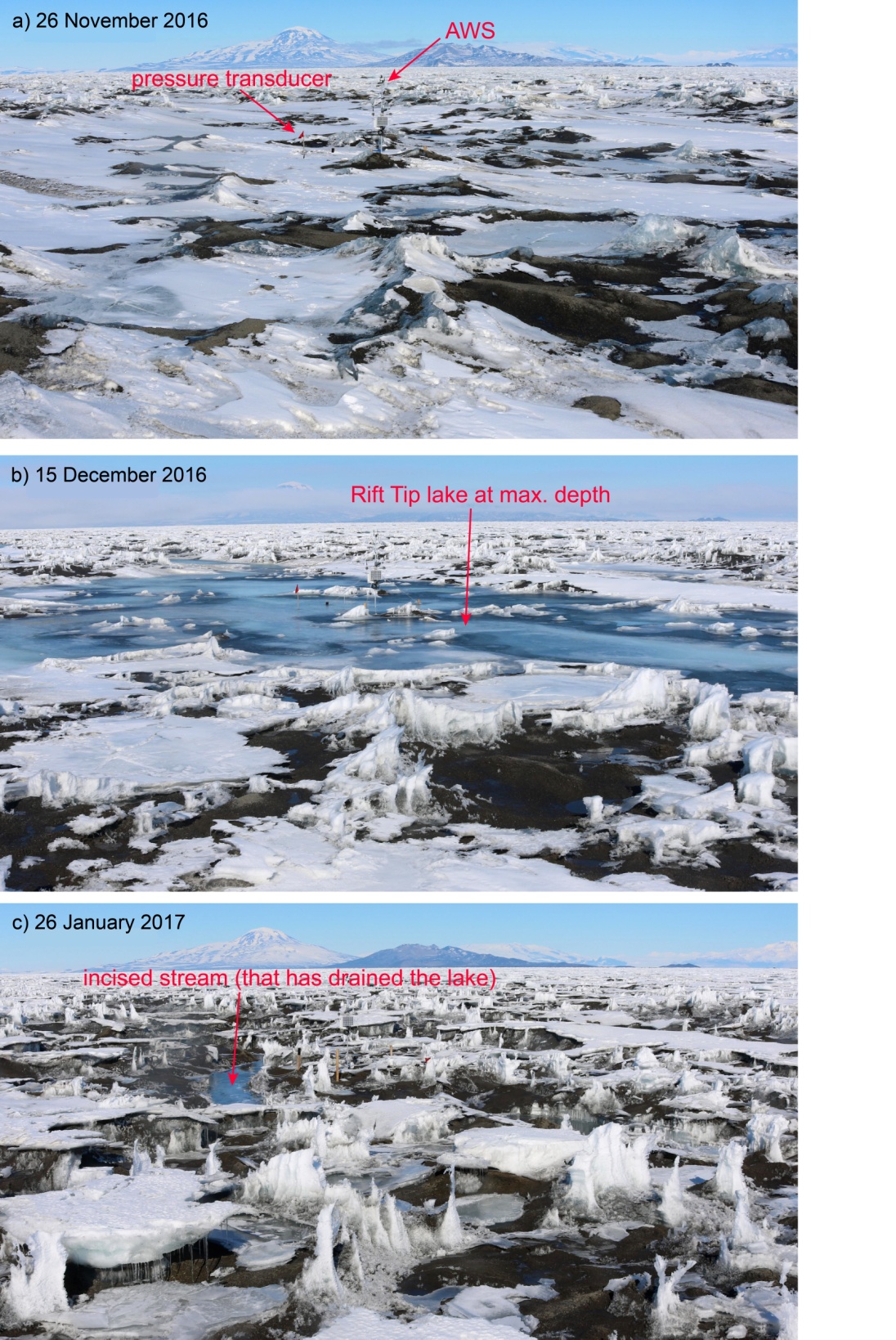
**

**Supplementary Fig. 1** Photographs of Rift Tip Lake before during and after it filled. These are three of the 3015 photos used to produce the movie of Rift Tip lake filling and draining (Supplementary Movie 1) dated: (**a**) 26 November 2016 (when the lake was yet to fill); (**b**) 15 December 2016 (when the lake was at its maximum depth); and (**c**) 26 January 2017 (after the lake had drained via an incised surface stream). All photos taken at Noon, local time. The camera was set up near to Rift Tip GPS 1 (Fig. 1b, filled blue circle). See Methods for camera set-up and image processing details.

**Supplementary Fig. 2** Vertical ice shelf deflection and stresses associated with the drainage of Rift Tip WT and Peanut lakes. (**a**) is for Rift Tip, (**b**) is for WT and (**c**) is for Peanut. In each subplot, the top panel shows vertical ice-shelf deflection (orange line) as a function of distance from lake centre, which is computed by an exact analytic solution for thin elastic plate flexure above seawater in response to the drainage of each lake. The total volumes of water unloaded from each lake are indicated on each subplot, and are equal to the calculated seasonal net meltwater budgets within a 250 m radius of each lake’s GPS 1 (Figs. 3b and 4). The results shown are the best matches between the analytic solutions and the measured deflections at each lake centre (i.e. GPS 1) with parameter values; *H* = 10 m and *E* = 1 GPa. *R* was varied for each lake to enable the simulated and measured deflections at each lake centre to match. Locations of GPSs 1, 2 and 3 at each lake site are indicated. The bottom panel of each subplot shows the associated stresses (radial (red line), azimuthal (blue line), and von-Mises (green line) as a function of distance from lake centres), which are all evaluated at the upper ice-shelf surface.


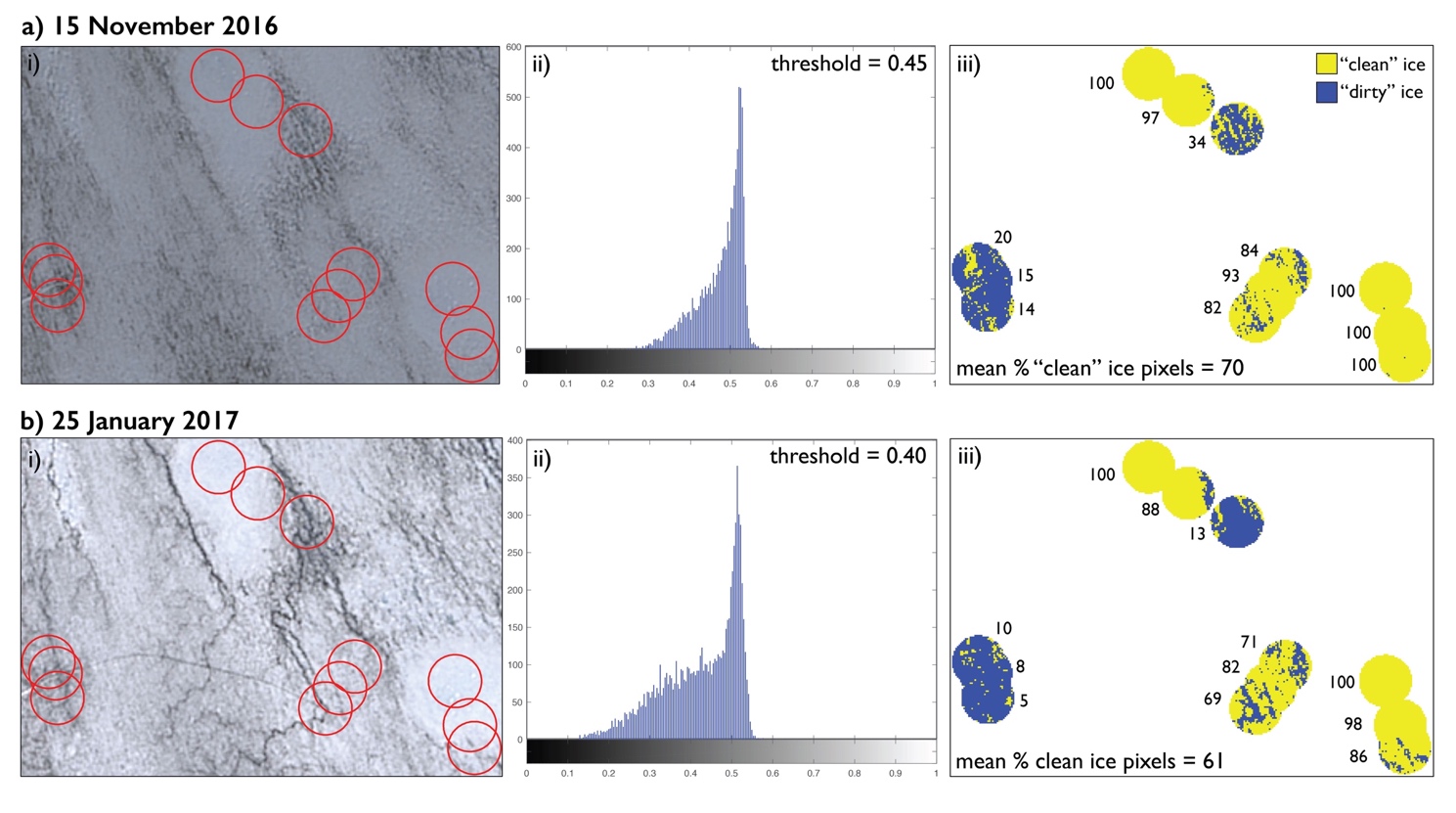


**Supplementary Fig. 3** Illustration of method used to apportion pixels as either clean or dirty for PDD melt calculations. All pixels in each of the 12 circles (*r* = 250 m) were apportioned as either “clean” or “dirty” ice for PDD melt calculations in each Landsat 8 image through the season. Examples are shown for: (**a**) 15 November 2016 and (**b**) 25 January 2017 (the first and last available Landsat 8 images in the melt season, respectively, which contain meltwater). (i) True colour Landsat 8 images with outlines of the circles. (ii) Histograms of pixel brightness (x-axis) versus number of pixels (y-axis) used to determine threshold values for dark versus light pixels in each image. (iii) Masks of “clean” (yellow) versus “dirty” (blue) ice within the 12 circles, based on the threshold determined for each image. Next to each circle, the percentage of clean ice pixels in each circle is stated. See Methods for further details.

| ***H* (m)** | ***R* (m)** | ***E* (GPa)** | **deflection at *r* = 0 (m)** | ***r* (m) where deflection < 0.01 m** | **max. *T*_vM_ within lake radius (kPa)** |
| --- | --- | --- | --- | --- | --- |
| 10 | 50 | 1 | 1.85 | 180 | 1858 |
| 15 | 50 | 1 | 1.09 | 280 | 1112 |
| 30 | 50 | 1 | 0.42 | 430 | 408 |
| **10** | **125** | **1** | **1.01** | **250** | **374** |
| 15 | 125 | 1 | 0.73 | 310 | 338 |
| 30 | 125 | 1 | 0.34 | 450 | 181 |
| 10 | 250 | 1 | 0.31 | 350 | 65 |
| 15 | 250 | 1 | 0.31 | 390 | 38 |
| 30 | 250 | 1 | 0.22 | 500 | 58 |
| 10 | 50 | 5 | 0.91 | 310 | 2717 |
| 15 | 50 | 5 | 0.52 | 400 | 1512 |
| 30 | 50 | 5 | 0.19 | 590 | 512 |
| 10 | 125 | 5 | 0.64 | 330 | 911 |
| 15 | 125 | 5 | 0.42 | 410 | 643 |
| 30 | 125 | 5 | 0.17 | 600 | 280 |
| 10 | 250 | 5 | 0.3 | 410 | 150 |
| 15 | 250 | 5 | 0.25 | 460 | 172 |
| 30 | 250 | 5 | 0.13 | 620 | 126 |
| 10 | 50 | 10 | 0.67 | 360 | 3105 |
| 15 | 50 | 10 | 0.37 | 460 | 1689 |
| 30 | 50 | 10 | 0.17 | 660 | 557 |
| 10 | 125 | 10 | 0.51 | 380 | 1205 |
| 15 | 125 | 10 | 0.31 | 470 | 794 |
| 30 | 125 | 10 | 0.13 | 670 | 322 |
| 10 | 250 | 10 | 0.28 | 440 | 262 |
| 15 | 250 | 10 | 0.21 | 520 | 264 |
| 30 | 250 | 10 | 0.1 | 680 | 161 |

**Supplementary Table 1** Sensitivity test results of the exact analytic solution for flexure of a thin elastic-plate in response to Ring Lake drainage. Values are varied for the three most sensitive parameters: Young’s Modulus (*E*), lake radius (*R*), and effective ice thickness (*H*) (see Methods for further details). Results are expressed as: vertical ice-shelf deflection at the lake centre (i.e. *r* = 0 m); distance from lake centre (*r*) where deflection <0.01 m; and maximum Von Mises stress (*T_vM_*) within the lake radius. The combination of parameter values that are found to produce the best match between the measured and modelled vertical ice-shelf deflection data are highlighted in bold font.

| **Date** | **Landsat Product ID** |
| --- | --- |
| 08 November 2016 | LC08_L1GT_054116_20161108_20170318_01_T2 |
| 15 November 2016 | LC08_L1GT_224128_20161115_20170318_01_T2 |
| 29 November 2016 | LC08_L1GT_226128_20161129_20170317_01_T2 |
| 03 December 2016 | LC08_L1GT_053116_20161203_20170317_01_T2 |
| 12 December 2016 | LC08_L1GT_221129_20161212_20170316_01_T2 |
| 24 December 2016 | LC08_L1GT_056115_20161224_20170315_01_T2 |
| 02 January 2017 | LC08_L1GT_055116_20170102_20170312_01_T2 |
| 11 January 2017 | LC08_L1GT_054116_20170111_20170311_01_T2 |
| 18 January 2017 | LC08_L1GT_224128_20170118_20170311_01_T2 |
| 25 January 2017 | LC08_L1GT_225128_20170125_20170311_01_T2 |

**Supplementary Table 2** Dates and IDs for the 10 cloud-free Landsat 8 images analysed in the study. These images were used to: i) estimate areas and depths, and therefore volumes, of ponded meltwater; and ii) apportion “dirty” versus “clean” pixels for calculations of meltwater production volumes, both plotted in Figs. 3 and 4. See Methods for further details.
