# Supplementary material for: Direct measurements of ice-shelf flexure caused by surface meltwater ponding and drainage
Source: Nat Commun. 2019 Feb 13;10:730. doi: 10.1038/s41467-019-08522-5 (PMC6374411; doi:10.1038/s41467-019-08522-5)
Supplement: Supplementary file 3 — Description of Additional Supplementary Files [file 41467_2019_8522_MOESM3_ESM.pdf]

Description of Additional Supplementary Files

Supplementary Movie 1: Movie of Rift Tip lake filling and draining. The movie was produced from 3015 photos, taken from near to Rift Tip GPS 1 (Fig. 1, filled blue circle) at 30-minute intervals from 25 November 2016 to 27 January 2017. See Methods for camera set-up and image processing details.
